# Relative Impact of Monotherapies for Vitiligo: A Network Meta‐Analysis Study

**DOI:** 10.1111/jocd.70078

**Published:** 2025-02-27

**Authors:** Aditya K. Gupta, Mary A. Bamimore, Julien Seneschal, Vincent Piguet, Mesbah Talukder

**Affiliations:** ^1^ Mediprobe Research Inc. London Ontario Canada; ^2^ Division of Dermatology, Department of Medicine, Temerty Faculty of Medicine University of Toronto School of Medicine Toronto Ontario Canada; ^3^ Department of Dermatology and Pediatric Dermatology, National Reference Center for Rare Skin Disorders, Hospital Saint‐André University of Bordeaux, CNRS UMR 5164, ImmunoConcept Bordeaux France; ^4^ Division of Dermatology Women's College Hospital Toronto Ontario Canada; ^5^ School of Pharmacy BRAC University Merul Badda Dhaka Bangladesh

## Abstract

**Background:**

Vitiligo, a stigmatizing condition characterized by patchy depigmented skin, has an estimated global prevalence of 0.36%. This condition is a risk factor for anxiety, depression, and even suicidal ideation. Hitherto, no network meta‐analysis has investigated the relative effect of vitiligo on relevant monotherapies.

**Aim:**

The current study determined the relative effect of monotherapies for vitiligo through network meta‐analyses (NMAs).

**Methods:**

The peer‐reviewed literature was systematically searched through PubMed and Scopus; studies that were eligible for quantitative analyses were those that were published in English and had an arm that investigated the effect of a monotherapy on vitiligo at 6 months. Studies of the randomized and observational designs were included.

**Results:**

The retrieved data were sufficient to analyze networks for phototherapy, Janus kinase inhibitors (JAKIs), calcineurin inhibitors, cyclosporine, corticosteroids, azathioprine, and minocycline. Modalities' effects, in each of the networks, were ranked with the surface under the cumulative ranking curve (SUCRA) metric; league tables were produced to depict agents' pairwise relative effects. Our secondary outcome was discontinuation due to any adverse event (AE) at 6 months.

**Conclusions:**

No significant differences are observed among JAK inhibitors; however, upadacitinib, cyclosporine, ritlecitinib, and dexamethasone are significantly more effective than minocycline. Psoralen (oral) + ultraviolet A (PUVA) and narrow band ultraviolet B (NB‐UVB) regimens show similar efficacy for repigmentation. Ruxolitinib 1.5% cream (once or twice daily), ruxolitinib 0.5% cream once daily, and ruxolitinib 0.15% cream once daily for 6 months do not differ significantly in efficacy. Mometasone furoate and tacrolimus 0.1% ointment are more effective than tacrolimus 0.03%.

## 1 Introduction

Vitiligo is a condition that is characterized by the occurrence of patchy depigmentation throughout the skin [1]; its global lifetime prevalence is estimated to be 0.36% [2]. The peer‐reviewed literature supports a positive correlation between having a diagnosis of vitiligo and developing depression [3], high anxiety [4], and even suicidal ideation [5, 6, 7, 8, 9]. Numerous therapies exist for this condition, and the effects thereof have been investigated in many studies [10, 11]. Oral and topical Janus kinase inhibitors (JAKIs) are among the recently developed treatment options for vitiligo. Evidence regarding the relative impact of vitiligo therapies is scant: many of the meta‐analyses to date [12, 13, 14, 15, 16, 17] are of the pairwise type; a network meta‐analysis (NMA) comparing numerous (i.e., 3 or more) therapies for this condition simultaneously has—to the best of our knowledge—never been published. The current study determined the relative effect of relevant monotherapies for vitiligo using NMAs.

## 2 Methods

The protocol for the current study was prospectively registered under the *International Platform Of Registered Systematic Review And Meta‐Analysis Protocols* (INPLASY) with the ID INPLASY2024110065 [18]. Our work was conducted in accordance with the Preferred Reporting Items for Systematic Reviews and Meta‐Analyses (PRISMA) guidelines [19].

### 2.1 Eligible Studies

The peer‐reviewed literature was systematically searched—through PubMed, Scopus, and reference mining—on October 21, 2024, to identify trial studies that were published in English and investigated the effect of relevant monotherapies with—Janus kinase inhibitors (JAKIs), ultraviolet (UV)‐based phototherapy, corticosteroids, calcineurin inhibitors, including cyclosporine, azathioprine, methotrexate, and minocycline—on vitiligo at 6 months from baseline. We included trials of randomized and observational designs: disconnected networks were connected using an approach supported by recommendations from Cameron (2006) and Leahy et al. (2019) [20, 21]; moreover, these recommendations have been followed in published peer‐reviewed studies [22, 23, 24]. Evidence quality was assessed for each study using Cochrane Collaboration's Risk of Bias 2 (RoB 2) [25] tool for randomized studies and ROBINS‐I (i.e., **R**isk **O**f **B**ias **I**n **N**on‐randomized **S**tudies‐**I**nterventions) [26] for observational studies.

### 2.2 Network Depiction

Monotherapies whose effects were compared in trials (i.e., direct evidence) were depicted through network plots, which the literature defines as a graph of ‘nodes’ and ‘edges’: the former corresponds to the respective therapy and the latter represents direct evidence (i.e., denotes comparison of effect from an actual head‐to‐head trial).

### 2.3 Outcomes

The relative effect of oral JAKIs, ultraviolet (UV)‐based therapies was analyzed in two separate networks; the relative effect of topical calcineurin inhibitors and topical JAKIs was analyzed in separate networks. Each NMA produced a surface under the cumulative ranking curve (SUCRA) value for each therapy; the value of SUCRA ranges between 0 (or 0%) and 1 (or 100%); it is a metric that ranks an intervention's effectiveness per outcome measure. League tables are 2‐dimensional tables that present the pairwise comparison of effect as per the outcome of interest. Relative effectiveness was the primary outcome of interest, while our secondary outcome of interest was relative safety—insofar as discontinuation due to any adverse event at 6 months. For each network, evaluation of demographic variables (i.e., age and sex distribution) was used to qualitatively assess that each network was transitive.

### 2.4 Network Meta‐Analyses

The extracted data were organized across spreadsheets, and analyses‐ready data were handled using *BUGSnet* [27] package in *RStudio* [28]. We ran Bayesian fixed effects NMAs with uniform priors. Our choice of the fixed‐effects model—as opposed to the random‐effects model—was based on Dettori et al.'s (2022) [29] explanation: though both models weight outcome data according to sample size, the weight of a smaller‐sized study under a random effects model is still larger than that under a fixed‐effects model [29]. Uniform priors were used with 20,000 iterations, 4 Markov Chain Monte Carlo (MCMC) chains, and 5000 adaptations.

## 3 Results

### 3.1 Identified Studies and Evidence Quality

**FIGURE 1:**
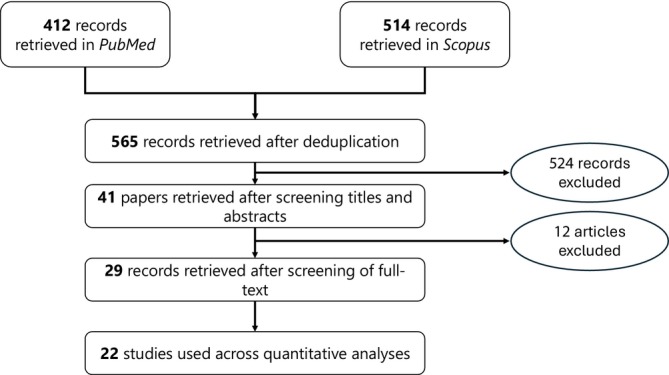
Schematic for the identification of included studies. Searches were made in PubMed and Scopus on October 21, 2024, to identify studies that will be eligible and included in quantitative analyses.

**FIGURE 2:**
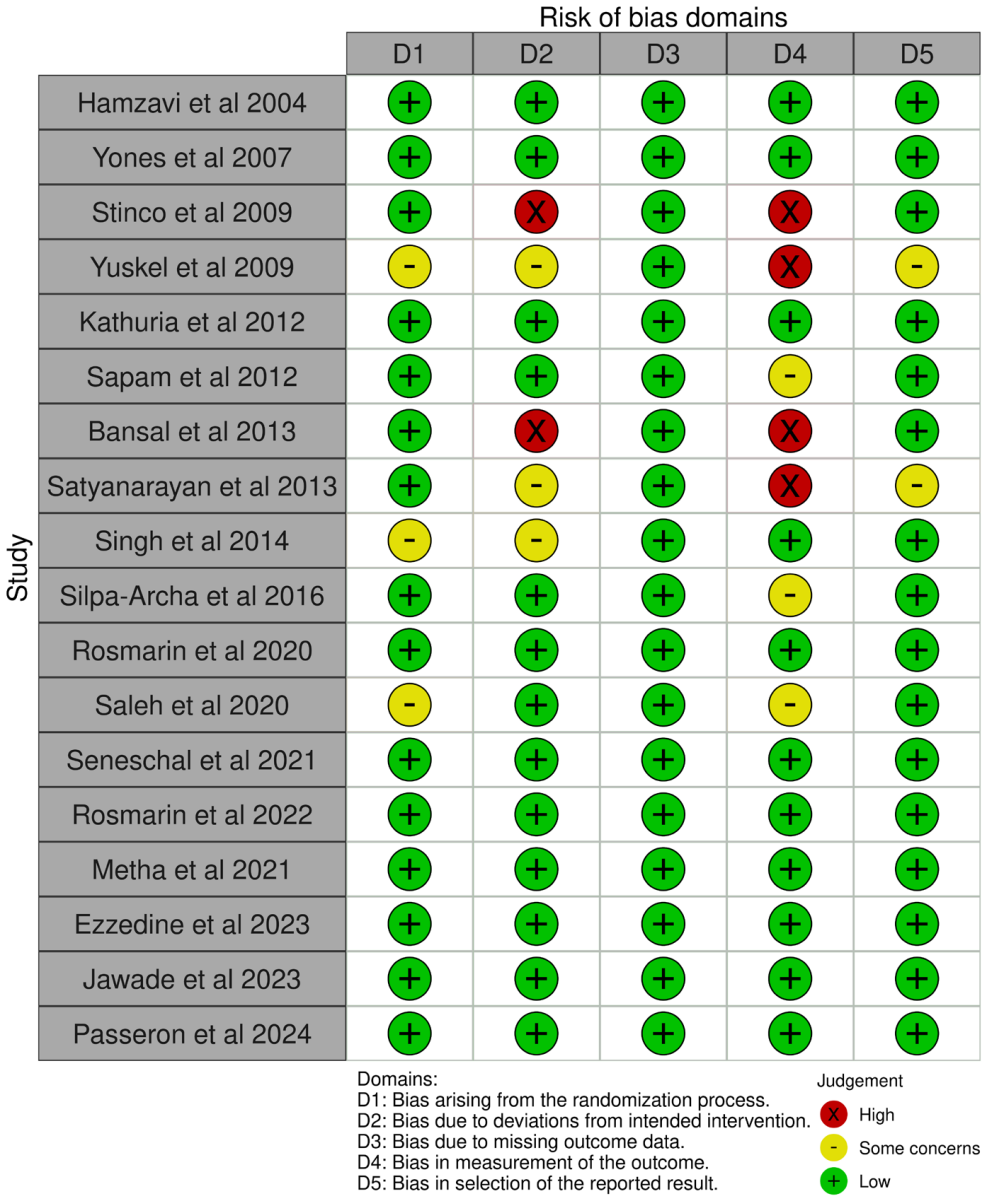
Qualitative Summary of Included Studies' Risk of Bias (Traffic plot for randomized studies).

**FIGURE 3:**
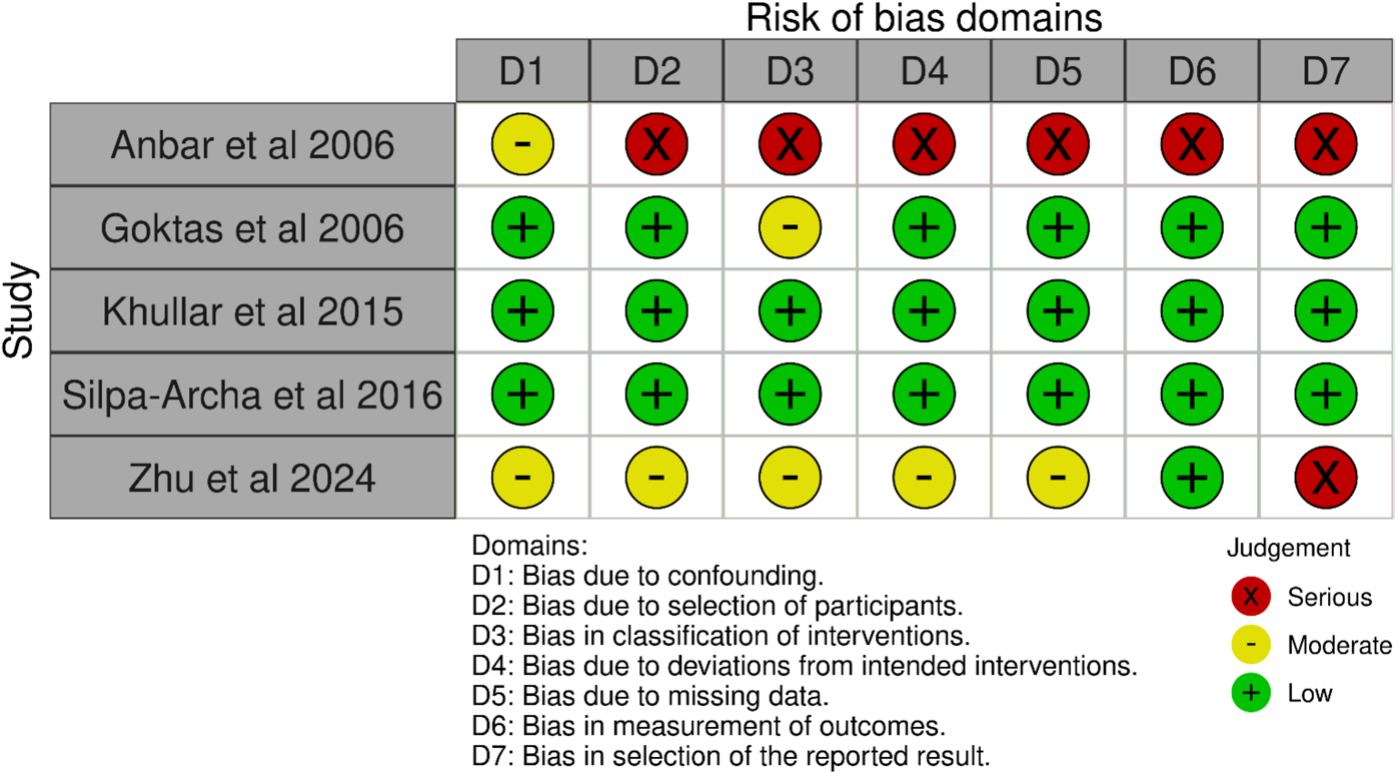
Qualitative Summary of Included Studies' Risk of Bias (Traffic plot for observational studies).

We identified a total of 22 studies; the schematic in Figure 1 summarizes the identification of the included studies. Summaries for our qualitative evaluation of evidence quality—as per study design (i.e., randomized vs. observational)—are presented in Figures 2 and 3, as well as Figures S1 and S2; a summary of included studies' characteristics is presented in Table 1.

**TABLE 1:** Summary of included studies' characteristics.

Author	Year	Study design	Agent	Regimen details	Age, years†	Sex	Sample size at baseline
Hamzavi et al. [30]	2004	Randomized (intra‐individual)	Narrow band Ultraviolet B	3 times a week for 6 months	47 (12.7)	M = 9, F = 13	22
Hamzavi et al. [30]	2004	Randomized (intra‐individual)	No light	3 times a week for 6 months	47 (12.7)	M = 9, F = 13	22
Anbar et al. [31]	2006	Observational	Narrow band Ultraviolet B	2 times a week for 6 months	24.52 (13.6)	M = 38, F = 100	150
Goktas et al. [32]	2006	Observational (intra‐individual)	Narrow band Ultraviolet B	3 times a week for 6 months	34.21 (11.65)	M = 11, F = 13	28
Yones et al. [33]	2007	Randomized	Psoralen + Ultraviolet A	2 times a week for 6 months	36 (Median)	M = 48%, F = 52%	25
Yones et al. [33]	2007	Randomized	Narrow band Ultraviolet B	2 times a week for 6 months	38 (Median)	M = 68%, F = 32%	25
Silpa‐Archa et al. [34]	2016	Randomized	Mometasone furoate 0.1% cream	2 times daily for 6 months	46.8 (15.60)	M = 1, F = 17	18
Silpa‐Archa et al. [34]	2016	Randomized	Tacrolimus 0.1% ointment	2 times a week for 6 months			
Metha et al. [35]	2021	Randomized	Cyclosporine 3 mg per kg	Once week for 4 months	29.92 (9.60)	M = 12, F = 13	25
Metha et al. [35]	2021	Randomized	Dexamethasone 2.5 mg	2 times per week for 4 months	32.2 (10.10)	M = 10, F = 15	25
Jawade et al. [36]	2023	Randomized	Azathioprine 50 mg OD	Once daily for 6 months	Not provided	Not provided	18
Singh et al. [37]	2014	Randomized	Minocycline 100 mg	Once daily for 6 months	35.2 (14.10)	M = 13, F = 12	25
Singh et al. [37]	2014	Randomized	Dexamethasone 2.5 mg	2 times per week for 6 months	25.96 (12.53)	M = 17, F = 8	25
Stinco et al. [38]	2009	Randomized	Narrow band Ultraviolet B	3 times a week for 6 months	48.8 (27 to 72)	M = 7, F = 6	13
Stinco et al. [38]	2009	Randomized	Tacrolimus 0.1% ointment	2 times daily for 6 months	43.2 (30 to 61)	M = 2, F = 14	16
Stinco et al. [38]	2009	Randomized	Pimecrolimus 1% cream	2 times daily for 6 months	42.9 (27 to 56)	M = 5, F = 10	15
Yuskel et al. [39]	2009	Randomized	Narrow band Ultraviolet B	3 times a week for 6 months	28 (18 to 67)	M = 8, F = 7	15
Kathuria et al. [40]	2012	Observational (intra‐individual)	Topical tacrolimus 0.1% ointment	2 times daily for 6 months	14 (5 to 55) Median)	M = 16, F = 12	29
Sapam et al. [41]	2012	Randomized	Psoralen + Ultraviolet A	3 times a week for 6 months	29.17 (11.228)	M = 10, F = 18	26
Sapam et al. [41]	2012	Randomized	Narrow band Ultraviolet B	3 times a week for 6 months	31.25 (10.26)	M = 9, F = 18	27
Bansal et al. [42]	2013	Randomized	Psoralen + Narrow band Ultraviolet B	3 times a week for 6 months	34.1 (12.6)	M = 8, F = 12	20
Bansal et al. [42]	2013	Randomized	Narrow band Ultraviolet B	3 times a week for 6 months	29.9 (14.5)	M = 11, F = 9	20
Satyanarayan et al. [43]	2013	Randomized (intra‐individual)	Narrow band Ultraviolet B	3 times a week for 6 months	14 to 36	M = 13, F = 12	25
Khullar et al. [44]	2015	Observational (intra‐individual)	Narrow band Ultraviolet B	3 times a week for 6 months	24.4 (8.6)	M = 20, F = 5	25
Silpa‐Archa et al. [34]	2016	Observational	Topical tacrolimus 0.1% ointment	2 times daily for 6 months	46.8 (15.60)	M = 1, F = 17	20
Rosmarin et al. [45]	2020	Randomized	Ruxolitinib 0.15% cream	once daily for 24 weeks	45.1 (11.5)	M = 13, F = 18	31
Rosmarin et al. [45]	2020	Randomized	Ruxolitinib 0.5% cream	once daily for 24 weeks	53.8 (14.3)	M = 19, F = 12	31
Rosmarin et al. [45]	2020	Randomized	Ruxolitinib 1.5% cream	once daily for 24 weeks	46.7 (11.7)	M = 11, F = 19	30
Rosmarin et al. [45]	2020	Randomized	Ruxolitinib 1.5% cream	twice daily for 24 weeks	49.5 (12.3)	M = 18, F = 15	33
Rosmarin et al. [45]	2020	Randomized	Vehicle		46.3 (13.1)	M = 12, F = 20	32
Saleh et al. [46]	2020	Randomized	Topical tacrolimus 0.03% ointment	2 times a week for 6 months	22 (14 to 47)	M = 10, F = 21	31
Seneschal et al. [47]	2021	Randomized	Topical tacrolimus 0.1% ointment	2 times daily for 6 months	47 (10.7)	M = 9, F = 11	20
Seneschal et al. [47]	2021	Randomized	Topical vehicle ointment	2 times daily for 6 months	48.4 (14.4)	M = 12, F = 10	22
Rosmarin et al. [48]	2022	Randomized	1.5% Ruxolitinib cream	2 times daily for 6 months	40.5 (15.4)	M = 85, F = 136	221
Rosmarin et al. [48]	2022	Randomized	Vehicle	2 times daily for 6 months	39.7 (16.7)	M = 59, F = 50	109
Ezzedine et al. [49]	2023	Randomized	Placebo	placebo	46.1 (11.5)	M = 26, F = 40	66
Ezzedine et al. [49]	2023	Randomized	Oral ritlecitinib	10 mg once daily for 24 weeks	46.6 (10)	M = 24, F = 25	49
Ezzedine et al. [49]	2023	Randomized	Oral ritlecitinib	30 mg once daily for 24 weeks	44.7 (13.5)	M = 22, F = 28	50
Ezzedine et al. [49]	2023	Randomized	Oral ritlecitinib	50 mg once daily for 24 weeks	43.3 (10.4)	M = 28, F = 39	67
Ezzedine et al. [49]	2023	Randomized	Oral ritlecitinib	100/50 mg once daily for 24 weeks	44.2 (11.2)	M = 36, F = 31	67
Ezzedine et al. [49]	2023	Randomized	Oral ritlecitinib	200/50 mg once daily for 24 weeks	45.4 (12.2)	M = 35, F = 30	65
Passeron et al. [50]	2024	Randomized	Oral upadacitinib	6 mg once daily for 24 weeks	45.1 (11.68)	M = 23, F = 26	49
Passeron et al. [50]	2024	Randomized	Oral upadacitinib	11 mg once daily for 24 weeks	45.4 (11.90)	M = 13, F = 34	47
Passeron et al. [50]	2024	Randomized	Oral upadacitinib	22 mg once daily for 24 weeks	48.2 (11.13)	M = 17, F = 26	43
Passeron et al. [50]	2024	Randomized	Placebo	placebo	46.8 (10.48)	M = 17, F = 29	46
Zhu et al. [51]	2024	Observational	Oral tofacitinib	5 mg once daily for 6 months	29 (7.87)	M = 2, F = 3	5
Zhu et al. [51]	2024	Observational	Oral baricitinib	2 mg once daily for 6 months	37.2 (13.75)	M = 3, F = 2	5
Zhu et al. [51]	2024	Observational	Oral upadacitinib	15 mg once daily for 6 months	38.2 (12.17)	M = 2, F = 3	5

† indicates standard deviation.

### 3.2 Phototherapy

For our network for phototherapy (Figure S3), psoralen (oral) + ultraviolet A (PUVA) 3 times a week for 6 months was more effective than narrow band ultraviolet B (NB‐UVB) 3 times a week for 6 months, which, in turn, was more effective than Narrow band Ultraviolet B 2 times a week for 6 months—in terms of the probability of achieving 25% or greater repigmentation after 6 months of therapy (Table 2). However, the league table in Figure S4 shows no relative difference in effectiveness across pairwise comparisons.

**TABLE 2:** Network for phototherapy: Occurrence of 25% or greater repigmentation at 6 months (SUCRA).

Regimen	SUCRA (%)
Psoralen + Ultraviolet A 3 times a week for 6 months	70.34
Narrow band Ultraviolet B 3 times a week for 6 months	59.58
Narrow band Ultraviolet B 2 times a week for 6 months	20.06

### 3.3 Oral Therapies

**FIGURE 4:**
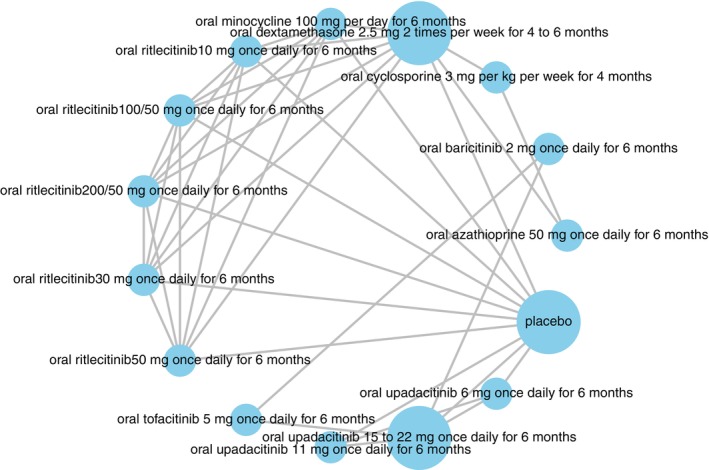
Network for oral JAKIs: Mean percent reduction in VASI at 6 months (network plot).

**FIGURE 5:**
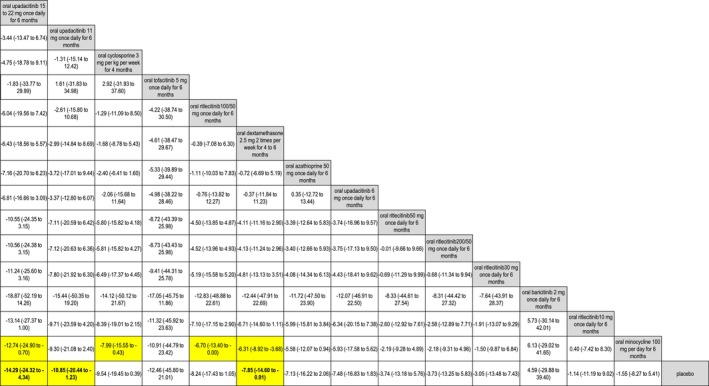
Network for oral JAKIs: Mean percent reduction in VASI at 6 months (league table).

Regarding our network for oral therapies (Figure 4), oral upadacitinib 15–22 mg once daily for 6 months was the most effective option, while placebo was the least effective option in terms of mean percent reduction in Vitiligo Area Scoring Index (VASI) at 6 months (Table 3). In terms of this outcome, oral upadacitinib 15–22 mg once daily for 6 months was significantly more effective than placebo (mean difference = −14.29%, 95% CI: −24.32%, −4.34%, *p* < 0.05) and oral minocycline 100 mg per day for 6 months (mean difference = −12.74%, 95% CI: −24.90%, −0.70%, *p* < 0.05) (Figure 5).

**TABLE 3:** Network for oral therapies: Mean percent reduction in VASI at 6 months (SUCRA).

Regimen	SUCRA (%)
Oral upadacitinib 15 to 22 mg once daily for 6 months	86.3
Oral upadacitinib 11 mg once daily for 6 months	73.62
Oral cyclosporine 3 mg per kg per week for 4 months	72.74
Oral tofacitinib 5 mg once daily for 6 months	65.69
Oral ritlecitinib 100/50 mg once daily for 6 months	65.25
Oral dexamethasone 2.5 mg 2 times per week for 4–6 months	64.71
Oral azathioprine 50 mg once daily for 6 months	57.5
Oral upadacitinib 6 mg once daily for 6 months	57.5
Oral ritlecitinib 50 mg once daily for 6 months	38.64
Oral ritlecitinib 200/50 mg once daily for 6 months	38.52
Oral ritlecitinib 30 mg once daily for 6 months	35.3
Oral baricitinib 2 mg once daily for 6 months	27.08
Oral ritlecitinib 10 mg once daily for 6 months	25.23
Oral minocycline 100 mg per day for 6 months	23.88
Placebo	17.99

### 3.4 Topical Therapy

We had two networks for topical therapy: (1) mean percent reduction in VASI at 6 months with oral JAKIs and (2) occurrence of 50% or greater repigmentation at 6 months of therapy with topical calcineurin inhibitors.

**FIGURE 6:**
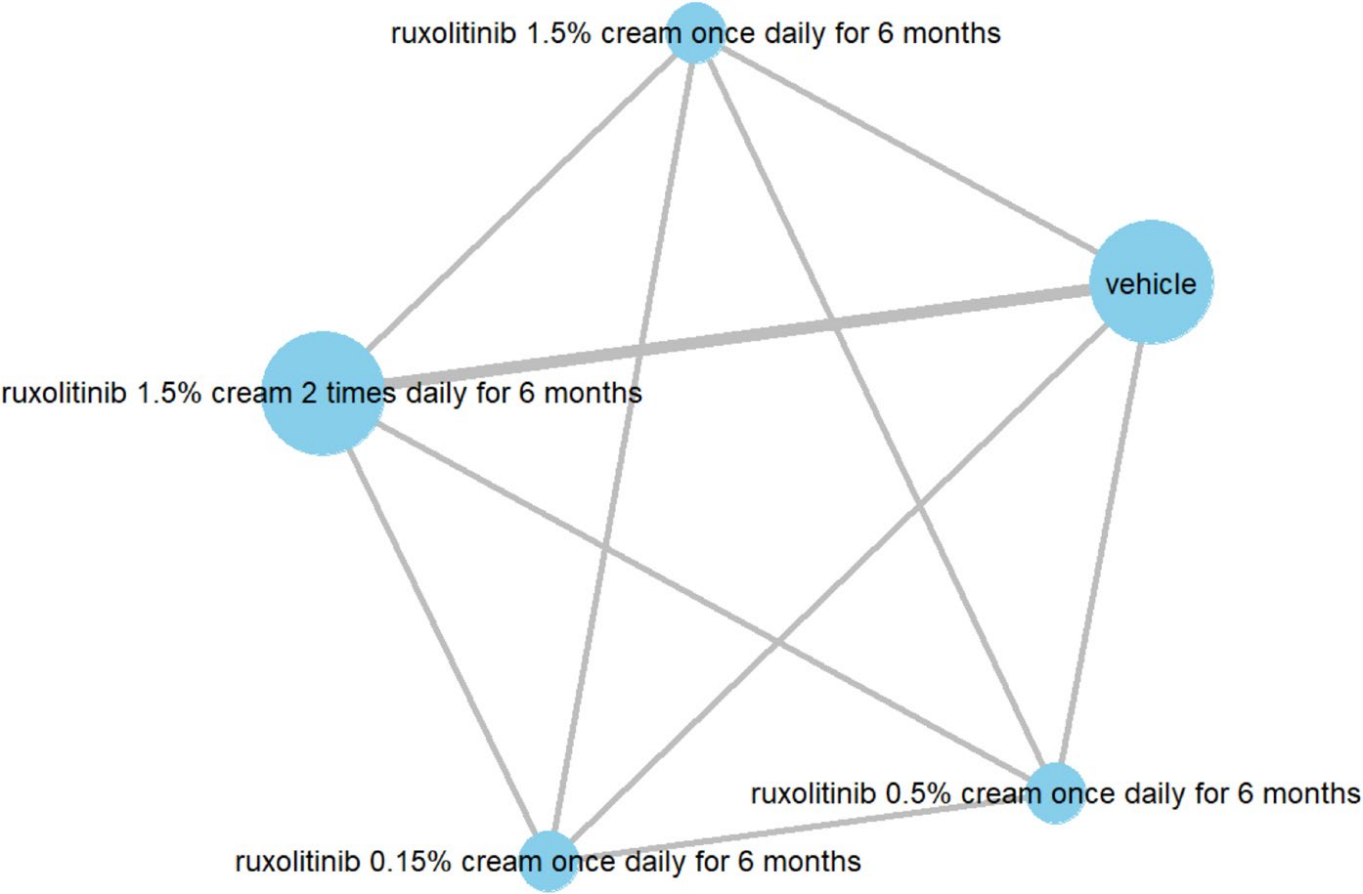
Network for topical therapy: Mean percent reduction in VASI at 6 months (network plot).

**FIGURE 7:**
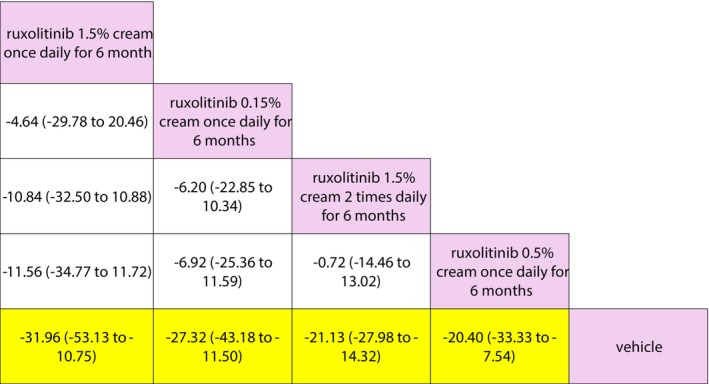
Network for topical therapy: Mean percent reduction in VASI at 6 months (league table).

For the first network for topical therapy (Figure 6), vehicle was the least effective, while ruxolitinib 1.5% cream once daily for 6 months was the most effective (Table 4). The only statistically significant pairwise difference in effect was observed between each of the active comparators (i.e., the non‐placebo options) versus placebo (Figure 7).

**TABLE 4:** Network for topical therapy: Mean percent reduction in VASI at 6 months (SUCRA).

Regimen	SUCRA (%)
Ruxolitinib 1.5% cream once daily for 6 months	82.81
Ruxolitinib 0.15% cream once daily for 6 months	72.16
Ruxolitinib 1.5% cream 2 times daily for 6 months	48.5
Ruxolitinib 0.5% cream once daily for 6 months	46.46
Vehicle	0.07

In the second network for topical therapy (Figure S5), mometasone furoate 0.1% cream two times daily for 6 months was more effective than topical tacrolimus 0.1% ointment two times daily for 6 months, which, in turn, was more effective than topical tacrolimus 0.03% ointment two times daily for 6 months, which, in turn, was more effective than vehicle—in terms of occurrence of 50% or greater repigmentation at 6 months (Table 5). There was no significant difference between mometasone furoate 0.1% cream two times daily for 6 months and topical tacrolimus 0.1% ointment two times daily for 6 months (Figure S6).

**TABLE 5:** Network for topical therapy: Occurrence of 50% or greater repigmentation at 6 months (SUCRA).

Regimen	SUCRA (%)
Mometasone furoate 0.1% cream 2 times daily for 6 months	98.96
Topical tacrolimus 0.1% ointment 2 times daily for 6 months	75.56
Topical tacrolimus 0.03% ointment 2 times a week for 6 months	43.46
Vehicle	17.44
Hydrocortisone acetate 1% ointment 2 times a week for 6 months	14.57

### 3.5 Safety

Our NMA showed that, in terms of discontinuation of therapy due to any AE with oral JAKIs, ‘oral upadacitinib 22 mg once daily for 6 months’ was ranked the least safe (Table 6).

**TABLE 6:** Network for relative safety of vitiligo monotherapies as per SUCRA for discontinuation due to any adverse event at 6 months, SUCRA.

Regimen	SUCRA (%)
Oral minocycline 100 mg per day for 6 months	91.5
Oral cyclosporine 3 mg per kg per week for 4 months	83.04
Oral azathioprine 50 mg once daily for 6 months	82.73
Oral upadacitinib 11 mg once daily for 6 months	82
Oral dexamethasone 2.5 mg 2 times per week for 4–6 months	74.6
Oral ritlecitinib 200/50 mg once daily for 6 months	48.15
Oral upadacitinib 6 mg once daily for 6 months	32.91
Control	32.17
Oral ritlecitinib 30 mg once daily for 6 months	32.03
Oral ritlecitinib 10 mg once daily for 6 months	31.43
Oral ritlecitinib 100/50 mg once daily for 6 months	31.16
Oral ritlecitinib 50 mg once daily for 6 months	23.9
Oral upadacitinib 22 mg once daily for 6 months	4.38

## 4 Discussion

To the best of our knowledge, no NMA has been published on the relative effectiveness of relevant monotherapies for vitiligo; through NMAs, the current study determined the relative effectiveness of JAKIs, UV‐based phototherapies, corticosteroids, calcineurin inhibitors, and minocycline—where agents' effect in each of the four networks were simultaneously compared using a Bayesian framework. Combination therapy was not compared because the potential ‘synergistic’ impact of polytherapy would make it hard to tease constituent monotherapies' effects.

According to the International Vitiligo Task Force [11], dermatologists currently suggest the use of oral JAKIs, topical JAKIs, topical calcineurin inhibitors, and so forth; this task force stated that oral PUVA is no longer recommended—due to supporting evidence regarding toxicity. Our NMA showed the effect of both NB‐UVB three and two times a week for 6 months to not be significantly different from oral PUVA three times a week for 6 months. Hence, our study supports these NB‐UVB therapies being equivalent alternatives to oral PUVA; however, given that available data allowed for only one outcome to be analyzed (occurrence of 25% or greater repigmentation at 6 months), future studies can investigate the relative effects of oral PUVA and NB‐UVB therapies on other relevant outcomes of vitiligo severity.

Our quantitative findings for oral ritlecitinib and oral tofacitinib are congruent with Sood et al.'s (2024) non‐quantitative syntheses of the evidence base: the authors [52] reported oral tofacitinib to be the most favorable oral JAKI [52], followed by oral ritlecitinib which, in turn, was more favorable than abrocitinib. The authors used data from Xu et al. (2024) [53] for comparisons with oral abrocitinib; we did not include Xu et al.'s (2024) results in the current work because this oral JAKI was used concomitantly with NB‐UVB; the current study only reviewed monotherapies. In congruence with Sood et al.'s (2024) work, we found oral tofacitinib 5 mg once daily for 6 months to be ranked higher than the various doses of oral ritlecitinib, in terms of mean percent reduction in VASI at 6 months—albeit our league table showed no statistically significant difference in relative effects (a finding that may be explained by low statistical power).

While the literature is filled with published studies on biologics' impact on vitiligo [54], we found none that could be used for our analyses. Many such studies were case reports and case series. Hence, our work supports the conduct of future randomized trials investigating the effect of monotherapy with monoclonal antibodies and interleukin inhibitors on vitiligo. Studies investigating methotrexate did not meet our eligibility criteria for our networks.

Our analyses did not compare monotherapies' impact insofar as the Vitiligo Disease Activity Score (VIDA) because studies that used VIDA did not meet our inclusion criteria. Notwithstanding that, we found more studies that used VASI than VIDA. Future trials may want to use VIDA to the same extent that VASI has been used hitherto. The conduct of more and more studies using multiple outcome measures besides VASI would also allow for various kinds of well‐powered subgroup analyses—such as subgroup analyses according to the site of vitiligo, type of vitiligo, duration of disease and specific demographic (e.g., a particular race).

The International Vitiligo Task Force also supports the use of topical tacrolimus. In our NMA, topical tacrolimus ointment 0.1% applied twice daily for 6 months had a higher SUCRA value than the tacrolimus 0.03% formulation used twice daily for 6 months; however, the difference was not significantly different between the two strengths (Table 5, Figure S5).

The conduct of additional blinded studies on vitiligo monotherapies would assist in expanding the knowledge base pertaining to the efficacy and safety of the oral and topical JAK inhibitors. Furthermore, additional blinded, randomized trials need to further investigate JAKIs like oral tofacitinib. Though we investigated this oral JAKI, the outcome data thereof was from a non‐blinded study. We also did not separate our NMAs according to different age groups because of lack of data availability.

Findings from our work would not only guide decision‐making but would also support the conduct of larger blinded, multi‐arm trials for the expansion of empirical data on therapies for vitiligo. The eventual acceptability of any of the newer therapies will be determined by factors such as safety, efficacy, route of administration (oral vs. topical. vs. Injection), regulatory approval status, and costing to the end user, that is, the patient.

## 5 Conclusion

Regarding phototherapy, there is no significant difference in efficacy among PUVA 3 times a week for 6 months, NB‐UVB 3 times a week for 6 months, and NB‐UVB two times a week for 6 months in terms of the probability of achieving 25% or greater repigmentation after 6 months of treatment.

For oral therapy, no significant difference in efficacy is observed between various JAKIs based on the mean percentage reduction in the VASI at 6 months. However, oral upadacitinib (15–22 mg once daily for 6 months), oral cyclosporine (3 mg/kg per week for 4 months), oral ritlecitinib (100/50 mg once daily for 6 months), and oral dexamethasone (2.5 mg twice per week for 4–6 months) show significantly different efficacy compared to oral minocycline (100 mg per day for 6 months).

Regarding topical therapy, in terms of vitiligo occurrence of 50% or greater: repigmentation at 6 months, mometasone furoate 0.1% cream (twice daily for 6 months) and topical tacrolimus 0.1% ointment (twice daily for 6 months) are significantly more effective than topical tacrolimus 0.03% ointment (twice a week for 6 months). With regard to mean reduction in VASI at 6 months, ruxolitinib 1.5% cream (once or twice daily), ruxolitinib 0.5% cream once daily, and ruxolitinib 0.15% cream once daily for 6 months do not differ significantly in efficacy.

Overall, the findings from this study provide valuable insights to inform the clinical care of vitiligo, highlighting key areas for improving patient management. Moreover, the results underscore the need for large, multi‐arm randomized controlled trials to validate these findings and explore comprehensive treatment strategies for vitiligo. Our findings serve as quantitative evidence on the relative efficacy of oral therapies on the mean reduction in VASI scores at 6 months.

## Author Contributions

Conception of the manuscript was done by M.T. and A.K.G. Data analysis was performed by M.A.B. The manuscript was drafted by M.A.B., A.K.G., and M.T., and was substantively edited and revised by J.S., V.P., A.K.G., and M.T.

## Ethics Statement

Approval from an ethics board was not required as there was no direct involvement with human participants.

## Conflicts of Interest

A.K.G., M.A.B., J.S., and M.T. declare no conflicts of interest. V.P. has received grants from AbbVie, Bausch Health, Celgene, Eli Lilly, Incyte, Janssen, LEO Pharma, L'Oréal, Novartis, Organon, Pfizer, Sandoz, and Sanofi, received payment or honoraria for speaking engagement from Sanofi China, participated on an advisory board for LEO Pharma, Novartis, Sanofi, and Union Therapeutics, and received equipment donation from L'Oréal. V.P. declares that the interests do not affect the objectivity or integrity of this article.

## Supporting information


**Figure S1.** Qualitative summary of included studies’ risk of bias (overall evaluation according to domain for randomized studies).
**Figure S2.** Qualitative summary of included studies’ risk of bias (overall evaluation according to domain for observational studies).
**Figure S3.** Network for phototherapy: vitiligo occurrence of 25% or greater: repigmentation at 6 months (network plot).
**Figure S4.** Network for phototherapy: vitiligo occurrence of 25% or greater: repigmentation at 6 months (league table).
**Figure S5.** Network for topical therapy: vitiligo occurrence of 50% or greater: repigmentation at 6 months (network plot).
**Figure S6.** Network for topical therapy: vitiligo occurrence of 50% or greater: repigmentation at 6 months (league table).

## Data Availability

Data can be made available upon reasonable request.
