# Supplementary material for: Relative Impact of Monotherapies for Vitiligo: A Network Meta‐Analysis Study
Source: J Cosmet Dermatol. 2025 Feb 27;24(3):e70078. doi: 10.1111/jocd.70078 (PMC11866277; doi:10.1111/jocd.70078)
Supplement: Supplementary file 1 — Figure S1. Qualitative summary of included studies’ risk of bias (overall evaluation according to domain for randomized studies). Figure S2. Qualitative summary of included studies’ risk of bias (overall evaluation according to domain for observational studies). Figure S3. Network for phototherapy: vitiligo occurrence of 25% or greater: repigmentation at 6 months (network plot). Figure S4. Network for phototherapy: vitiligo occurrence of 25% or greater: repigmentation at 6 months (league table). Figure S5. Network for topical therapy: vitiligo occurrence of 50% or greater: repigmentation at 6 months (network plot). Figure S6. Network for topical therapy: vitiligo occurrence of 50% or greater: repigmentation at 6 months (league table). [file JOCD-24-e70078-s001.pdf]

Supplementary Figure 1

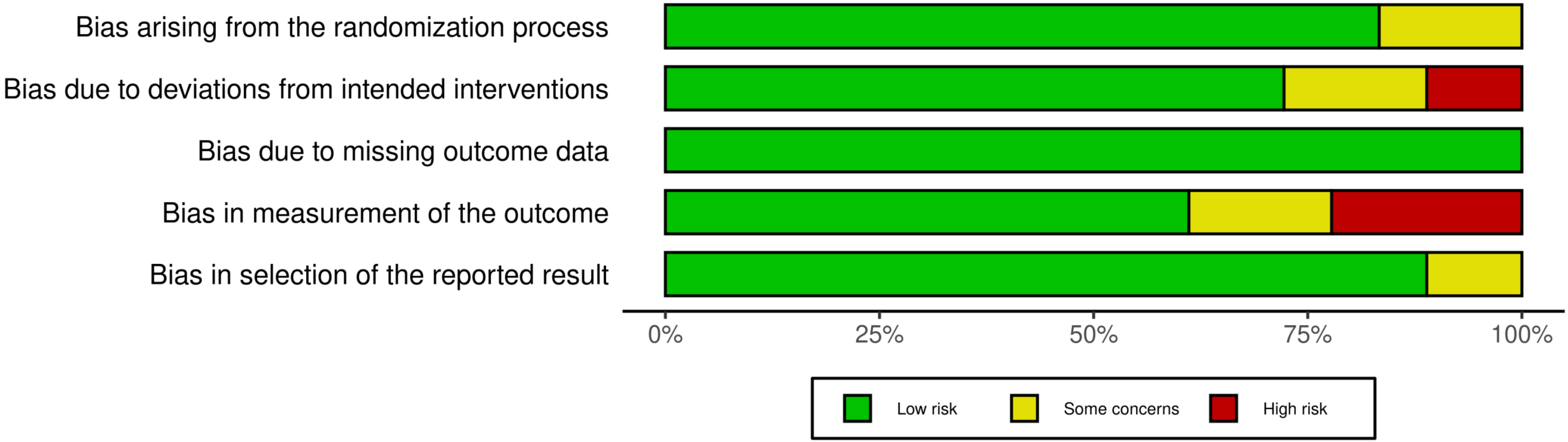

Supplementary Figure 1: Qualitative Summary of Included Studies' Risk of Bias (Overall evaluation according to domain for randomized studies)

Supplementary Figure 2

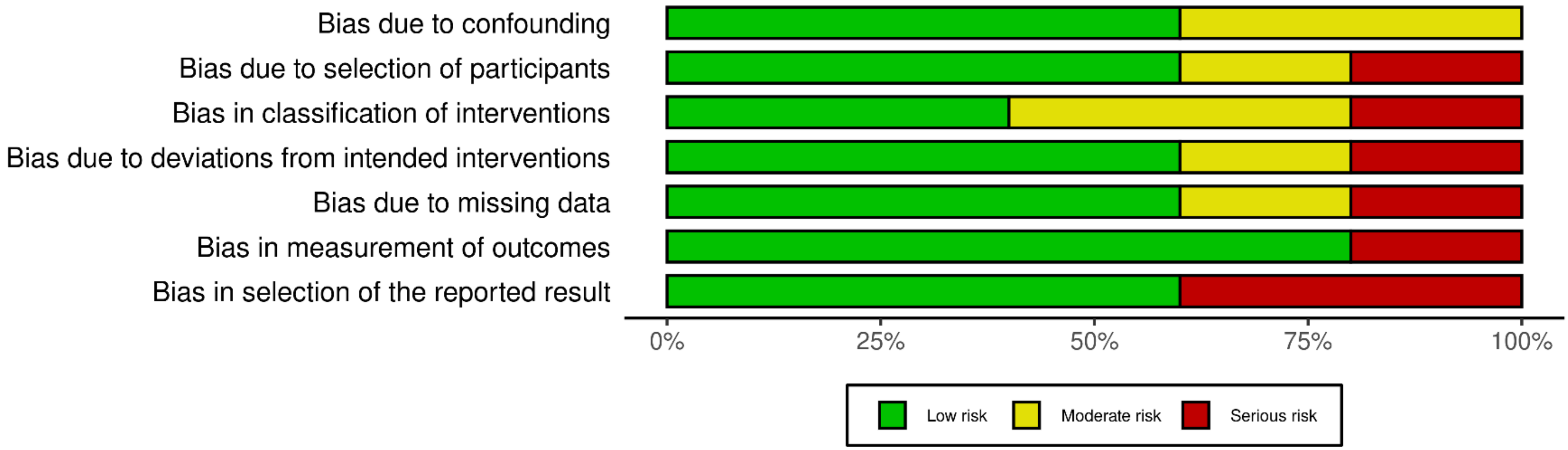

Supplementary Figure 2. Qualitative Summary of Included Studies' Risk of Bias (Overall evaluation according to domain for observational studies)

Supplementary Figure 3

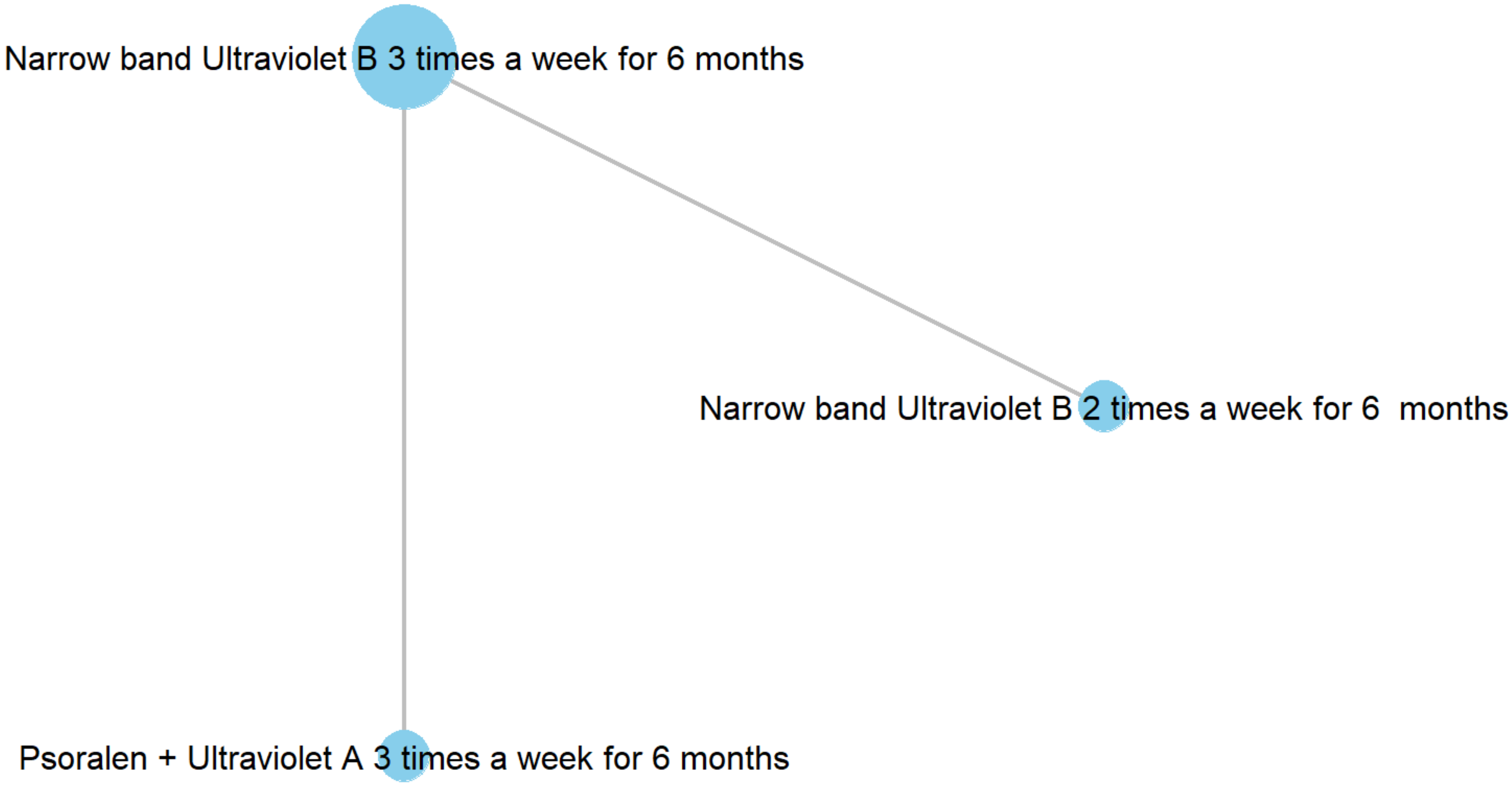

Supplementary Figure 3. Network for phototherapy: vitiligo occurrence of 25% or greater: repigmentation at 6 months (network plot)

Supplementary Figure 4

**psoralen + Ultraviolet A 3 times a week for 6 months**		
1.04 (0.81 to 1.31)	**narrow band Ultraviolet B 3 times a week for 6 months**	
1.32 (0.69 to 2.17)	1.28 (0.70 to 1.98)	**narrow band Ultraviolet B 2 times a week for 6 months**

Supplementary Figure 4. Network for phototherapy: vitiligo occurrence of 25% or greater: repigmentation at 6 months (league table)

Supplementary Figure 5

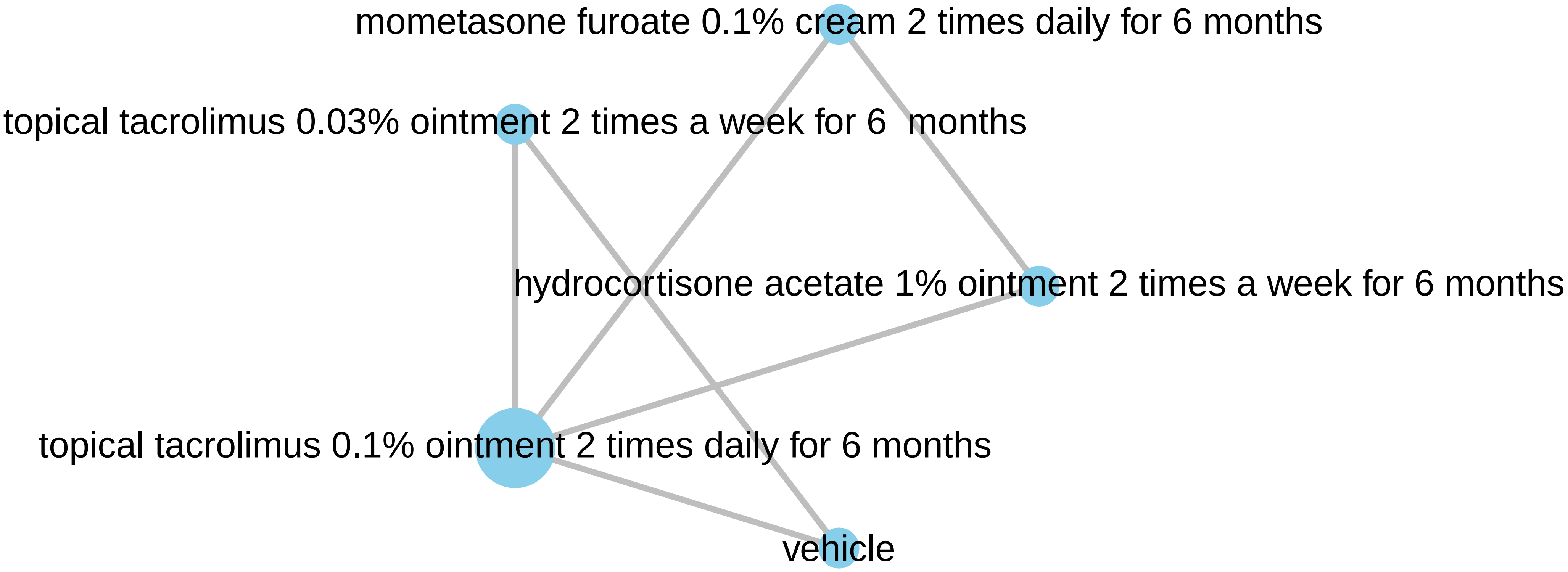

Supplementary Figure 5. Network for topical therapy: vitiligo occurrence of 50% or greater: repigmentation at 6 months (network plot)

Supplementary Figure 6. Network for topical therapy: vitiligo occurrence of 50% or greater: repigmentation at 6 months (league table)

Supplementary Figure 6

mometasone furoate 0.1% cream 2 times daily for 6 months				
2.87 (0.90 to 7.99)	topical tacrolimus 0.1% ointment 2 times daily for 6 months			
**8.47 (1.98 to 26.68)**	**2.95 (1.44 to 5.83)**	topical tacrolimus 0.03% ointment 2 times a week for 6 months		
**21.68 (3.31 to 84.60)**	**7.60 (2.20 to 23.57)**	2.86 (0.67 to 9.34)	vehicle	
**372.46 (3.51 to 957.43)**	**153.32 (1.13 to 417.26)**	58.08 (0.35 to 160.61)	21.14 (0.12 to 81.02)	hydrocortisone acetate 1% ointment 2 times a week for 6 months
